# lncRNA expression profiles related to apoptosis and autophagy in peripheral blood mononuclear cells of patients with rheumatoid arthritis

**DOI:** 10.1002/2211-5463.12913

**Published:** 2020-07-22

**Authors:** Jianting Wen, Jian Liu, Hui Jiang, Lei Wan, Ling Xin, Yue Sun, Pingheng Zhang, Yanqiu Sun, Ying Zhang, Xinlei Du, Xin Wang, Jie Wang

**Affiliations:** ^1^ Anhui University of Traditional Chinese Medicine Hefei China; ^2^ Department of Rheumatology and Immunology First Affiliated Hospital of Anhui University of Traditional Chinese Medicine Hefei China; ^3^ Institute of Rheumatology Anhui College of Traditional Chinese Medicine Hefei China; ^4^ Zhujiang Hospital Southern Medical University Guangzhou China

**Keywords:** apoptosis, autophagy, lncRNAs, rheumatoid arthritis, RNA‐seq

## Abstract

Long noncoding RNAs (lncRNAs) are >200‐bp molecules that do not generally code for proteins. Human lncRNAs have well‐characterized roles in gene expression regulation, particularly with regard to protein‐coding genes, and their dysregulation has been linked to disease. Here, we set out to investigate changes in the expression of lncRNAs related to apoptosis and autophagy in the peripheral blood mononuclear cells (PBMCs) of rheumatoid arthritis (RA). In addition, we aimed to correlate lncRNA expression profiles with clinical indexes and self‐perception of patients (SPP). To this end, we employed RNA sequencing of lncRNAs in PBMCs from three patients with RA and three healthy controls. We used bioinformatics to screen several dysregulated lncRNAs related to apoptosis and autophagy. To validate key lncRNA candidates, we performed quantitative reverse transcriptase–PCR on 20 patients with RA and 20 healthy controls. We found the expression of seven lncRNAs (MAPKAPK5‐AS1, ENST00000619282, C5orf17, LINC01189, LINC01006, DSCR9 and MIR22HG) was significantly altered in PBMCs of patients with RA. Receiver operating characteristic curve analysis suggested that MIR22HG [area under the curve (AUC) = 0.846, *P* = 0.000], DSCR9 (AUC = 0.783, *P* = 0.005), LINC01189 (AUC = 0.677, *P* = 0.034), MAPKAPK5‐AS1 (AUC = 0.644, *P* = 0.025) and ENST00000619282 (AUC = 0.636, *P* = 0.043) are potential biomarkers of RA. Spearman's correlation analysis revealed selected lncRNAs correlated with clinical indexes and SPP. Therefore, we highlight that some lncRNAs related to apoptosis and autophagy may serve as potential biomarkers for diagnosis and monitoring of RA progression, which also correlate with several clinical indexes and SPP.

## Graphical abstract

We investigated changes in the expression of lncRNAs related to apoptosis and autophagy in the peripheral blood mononuclear cells (PBMCs) of rheumatoid arthritis (RA). Expression of seven lncRNAs (MAPKAPK5‐AS1, ENST00000619282, C5orf17, LINC01189, LINC01006, DSCR9, and MIR22HG) was significantly altered in PBMCs of RA patients. Squares, triangles, and circles represent lncRNAs, miRNAs, and mRNAs, respectively. Red nodes: upregulation; blue nodes: downregulation.

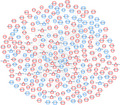

**Abbreviations**
AUC: area under the curve
C: complement
DEG: differentially expressed gene
GO: Gene Ontology
IgA: immunoglobulin A
IgG: immunoglobulin G
IgM: immunoglobulin M
KEGG: Kyoto Encyclopedia of Genes and Genomes
lncRNA: long noncoding RNA
MH: mental health
PBMC: peripheral blood mononuclear cell
PF: physical function
RA: rheumatoid arthritis
RE: role emotional
RF: rheumatoid factor
ROC: receiver operating characteristic
RP: role physical
qRT‐PCR: quantitative reverse transcriptase–PCR
SAS: Anxiety Self‐Assessment Scale
SD: standard deviation
SDS: Depression Self‐Assessment Scale
SF: social function
SPP: self‐perception of patients
VAS: Visual Analogue Scale
VT: vitality

Rheumatoid arthritis (RA) is a chronic inflammatory autoimmune disorder that affects peripheral joints and causes injury to secondary organs, including interstitial lung tissue and cardiac tissue [1, 2]. Misdiagnosis of RA can cause joint deformity and loss of ability to work [3]. However, the mechanism underlying the pathogenesis of RA is yet to be elucidated [4, 5]. Many studies have reported that apoptosis and autophagy play an important role in the pathogenesis of RA [6]. Early diagnosis and proper treatment are critical for improving the quality of life of patients and their families [7]. Nevertheless, current diagnostic methods suggest that the early diagnosis and treatment of RA remain a challenge [8, 9]. Therefore, new biomarkers are needed for improving the treatment and prognosis of RA [10].

More than 80% of the human genome is transcribed into RNA transcripts with little or no protein‐coding ability [11]. However, recent studies have revealed that although long noncoding RNAs (lncRNAs) do not generally code for proteins [12], they play a role in regulating the expression of human protein‐coding genes [13, 14]. Numerous studies have revealed that lncRNAs participate in the initiation and development of multiple diseases, including various cancers [15, 16], gynecological diseases [17, 18] and various autoimmune disorders [19, 20]. They act as miRNA sponges by sequestering miRNAs through base pair complementarity, thus keeping them away from their target mRNAs [21]. A recent study based on microarray analysis reported that the lncRNA ENST00000456270 was highly expressed in the peripheral blood mononuclear cells (PBMCs) of patients with RA and may serve as a potential biomarker for the diagnosis of RA [22]. Although the study demonstrated that lncRNAs are important biological molecules for studying the molecular mechanisms underlying the pathogenesis of RA, different aspects, such as apoptosis, autophagy, immune inflammation and oxidative stress, must be explored to gain a comprehensive understanding of the pathogenesis mechanism.

The objective of this study was to investigate the role of lncRNAs associated with apoptosis and autophagy as serological biomarkers for RA. We believe that our study will contribute to the development of effective strategies for the early diagnosis, treatment and prevention of RA.

## Materials and methods

### Sample collection

In this study, 20 patients with RA who were diagnosed at the Department of Rheumatology and Immunology in the First Affiliated Hospital of Anhui University of Traditional Chinese Medicine from November 2019 to December 2019 were included in the RA group. In addition, 20 age‐ and sex‐matched healthy subjects who underwent routine physical examinations in the same hospital during the same period served as the healthy control group (Table 1). All patients with RA fulfilled the criteria proposed by the American College of Rheumatology [23]. Patients with severe liver or kidney dysfunction, patients receiving immunosuppressive agents and pregnant women were excluded from the study. The research protocol was in accordance with the tenets of the Declaration of Helsinki and was approved by the Ethics Committee of First Affiliated Hospital of Anhui University of Traditional Chinese Medicine, and all patients signed an informed consent form.

**Table 1:** Characteristics and clinical features of RA patient samples used in RNA sequencing. F, female; M, male; NA, Not Applicable.

Index	RA (*n* = 20)	Control (*n* = 20)	*P*
Sex (M/F)	4/16	4/16	1.000
Age (years)^a^	55.4 ± 14.50	54.12 ± 13.29	0.872
Silk time (years)	10.02 ± 9.80	NA	NA
Erythrocyte sedimentation rate (mm·h^−1^)	46.65 ± 17.51	NA	NA
High‐sensitivity C‐reactive protein (mg·L^−1^)	21.38 ± 19.51	NA	NA
RF (U·mL^−1^)	132.77 ± 114.43	NA	NA
Cyclic citrullinated peptide (U·mL^−1^)	264.32 ± 265.03	NA	NA
IgA (g·L^−1^)	3.04 ± 0.52	NA	NA
IgG (g·L^−1^)	13.54 ± 3.34	NA	NA
IgM (g·L^−1^)	1.64 ± 0.72	NA	NA
C3 (g·L^−1^)	1.03 ± 0.15	NA	NA
C4 (g·L^−1^)	0.23 ± 0.10	NA	NA
Disease Activity Score in 28 joints score	6.46 ± 1.04	NA	NA
VAS score	6.89 ± 1.30	NA	NA
SAS score	57.86 ± 4.03	NA	NA
SDS score	56.80 ± 6.67	NA	NA
Physical function score	48.00 ± 13.17	NA	NA
Role physical score	15.00 ± 12.25	NA	NA
Body pain score	56.70 ± 13.21	NA	NA
General health score	45.25 ± 8.29	NA	NA
Vitality score	40.80 ± 7.16	NA	NA
SF score	51.88 ± 13.85	NA	NA
RE score	31.67 ± 24.67	NA	NA
MH score	48.60 ± 11.42	NA	NA

^a^ Mean ± SD. The significance difference between two groups was tested via analysis of Student's *t*‐test.

### Biochemical measurements

The clinical indexes were sex, age, course of disease, erythrocyte sedimentation rate, high‐sensitivity C‐reactive protein, rheumatoid factor (RF), anti‐cyclic citrullinated peptide Ig, immunoglobulin A (IgA), immunoglobulin G (IgG), immunoglobulin M (IgM), complement (C) 3 (C3) and C4. Self‐perception of patients (SPP) was measured using the following tools: Disease Activity Score in 28 joints, Visual Analogue Scale (VAS), Anxiety Self‐Assessment Scale (SAS) and Depression Self‐Assessment Scale (SDS) scores. The 36‐Item Short Form Health Survey was composed of eight dimensions: physical function, role physical, body pain, general health, vitality, social function (SF), role emotional (RE) and mental health (MH). All of the participants enrolled were asked to fill in the 36‐Item Short Form Health Survey scale under the guidance of two clinical doctors.

### RNA sequencing and bioinformatics analysis

Total RNA from each sample was used to construct cDNA libraries using the VAHTSTM Total RNA‐Seq (H/M/R) for high‐throughput sequencing. In brief, the RNA was reverse transcribed into first‐strand cDNA, followed by second‐strand cDNA synthesis, end repair, dA tailing and adaptor ligation. The digested products were purified using VAHTSTM DNA Clean Beads, and PCR amplified and sequenced using Illumina HiSeq 2500 (San Diego, CA, USA). The tagged cDNA libraries were paired‐end sequenced using an Illumina HiSeq 2500 with 51 plus 7 cycles by Dianxi Biotechnology, Shanghai, China. To fully understand the biological functions of lncRNAs related to immunity and inflammation, we performed Gene Ontology (GO) and Kyoto Encyclopedia of Genes and Genomes (KEGG) pathway enrichment analysis. Coexpression network analysis was performed using Comparative Co‐Expression Network Construction and Visualization Tool (CoExpNetViz, Chicago, IL, USA).

### PBMC preparation and total RNA extraction

PBMCs were isolated by Ficoll density centrifugation of 5 mL of blood layered on 6 mL of 1.077 g·mL^−1^ Ficoll‐Paque PLUS (GE Healthcare, Uppsala, Sweden). Cell concentration was adjusted between 5 × 10^6^ and 7 × 10^6^ cells·mL^−1^. Total RNA was extracted from PBMCs using TRIzol reagent (Invitrogen) according to the manufacturer's instructions. The quantity of RNA was measured using a NanoDrop spectrophotometer (NanoDrop Technologies, Wilmington, NC, USA).

### Quantitative reverse transcriptase–PCR analysis

cDNA was synthesized using the RT reagent kit with gDNA Eraser (TaKaRa, Shiga, Japan). The primers used in quantitative reverse transcriptase–PCR (qRT‐PCR) are shown in Table 2. β‐Actin expression was used as an internal control. Relative expression values were calculated for each of the three experiments as 2^−ΔΔCT^.

**Table 2:** Specific lncRNA primers used for qRT‐PCR analysis. F, forward; R, reverse.

Gene name	Sequence (5′–3′)	bp
*GAPDH*	F: GGAGCGAGATCCCTCCAAAAT	500
	R: GGCTGTTGTCATACTTCTCATGG	
*MAPKAPK5‐AS1*	F: GCGGAAAGTGACCAAGAG	2390
	R: CTTCTCCAGAGCCTGGTCAC	
*ENST00000619282*	F: CCTGGTGGGAGAGAATTGAA	651
	R: ATGAGAGCCAAGCAAGAGGA	
*C5orf17*	F: CCACACCGACACCTATACCC	2003
	R: TCGACTCTCCACTGTGATGC	
*LINC01189*	F: GTCTGCCCAGCTACTCCAAG	1193
	R: CTCCTACCGCTCCTGTTGAG	
*LINC01006*	F: GTGTGTCAGGCATTGTACCG	3350
	R: GCCCTGTTTCCAAAAGATCA	
*DSCR9*	F: ATTCCCTCCCCTATCACCAG	1681
	R: CCTAGCATACGCTGGAGGAC	
*MIR22HG*	F: TGGAGGAGGGGGTTAGAGTT	2659
	R: GGGGATCACATACCACCTTG	

### Statistical analysis

All data are presented as the means ± standard deviation (SD). Comparison between two groups was analyzed by the Student's *t*‐test or nonparametric Mann–Whitney–Wilcoxon test. The difference among more than two groups was analyzed by the one‐way ANOVA, followed by Tukey's *post hoc* test. A *P* value <0.05 was considered statistically significant. All statistical analyses were performed using the graphpad prism 8.0 (GraphPad Software Inc., San Diego, CA, USA) and spss 19.0 software (SPSS, Chicago, IL, USA).

## Results

### Screening of abnormal expression of lncRNAs in PBMCs from patients with RA and healthy controls

**Fig. 1:**
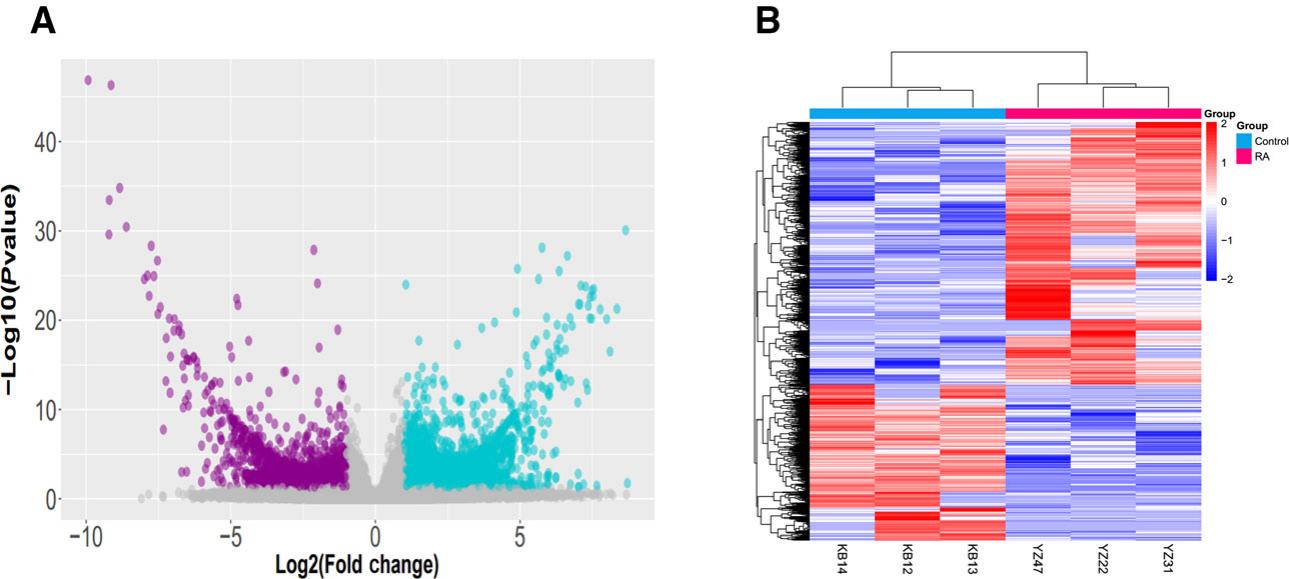
Expression profiling of lncRNAs. (A) Differentially expressed lncRNAs between patients with RA and healthy controls are displayed by volcano plots. Differentially expressed lncRNAs between two groups were compared by Student's *t*‐test. (B) Each column represents PBMCs from a patient with RA or a healthy control. The red lines represent up‐regulated lncRNAs.

We performed high‐throughput sequencing of lncRNAs in PBMCs from three patients with RA (KB12, KB13 and KB14) and three healthy controls (YZ22, YZ31 and YZ47). A total of 341 lncRNAs were detected. The volcano plots in Fig. 1A show the variations in the expression of lncRNAs related to apoptosis and autophagy between patients with RA and healthy controls. Hierarchical clustering further revealed differential expression of lncRNAs related to apoptosis and autophagy between patients with RA and healthy controls (Fig. 1B)

**Fig. 2:**
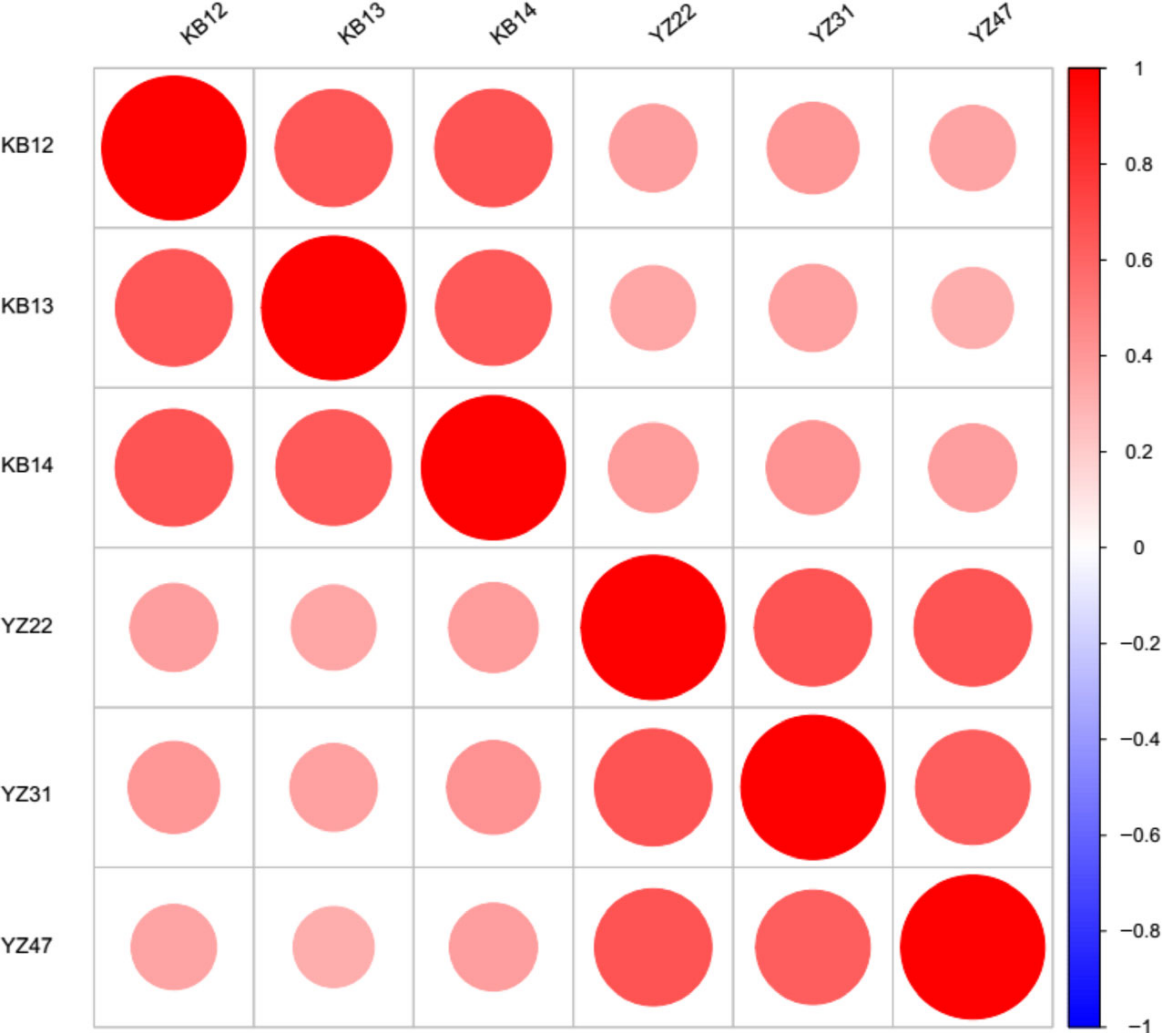
Correlation matrix showing Pearson's coefficients between the samples. Red represents positive correlation, and blue represents negative correlation; the larger the circle, the stronger the correlation. YZ (21, 22, 47) represents three healthy control samples, and KB (13, 12, 14) represents three RA samples.

The correlation matrix revealed very poor correlation between the samples (Fig. 2).

The top two up‐regulated and five down‐regulated lncRNAs are presented in Table 3.

**Table 3:** Basic characteristics of the seven differentially expressed lncRNAs.

lncRNAs	Gene ID	*P*	Fold change	Regulation	Gene symbol
MAPKAPK5‐AS1	51275	0.022	−2.50	Down‐regulation	TMEM116
ENST00000619282	ENST00000619282.1	0.000	1.80	Up‐regulation	P2RX7
C5orf17	439936	0.028	−2.01	Down‐regulation	NBPF14
LINC01189	643648	0.044	−2.16	Down‐regulation	ACSL1
LINC01006	100506380	0.030	−2.48	Down‐regulation	RNF32
DSCR9	257203	0.039	−1.94	Down‐regulation	TTC3
MIR22HG	84981	0.031	1.66	Up‐regulation	SMYD4

### Bioinformatics analysis of lncRNAs

**Fig. 3:**
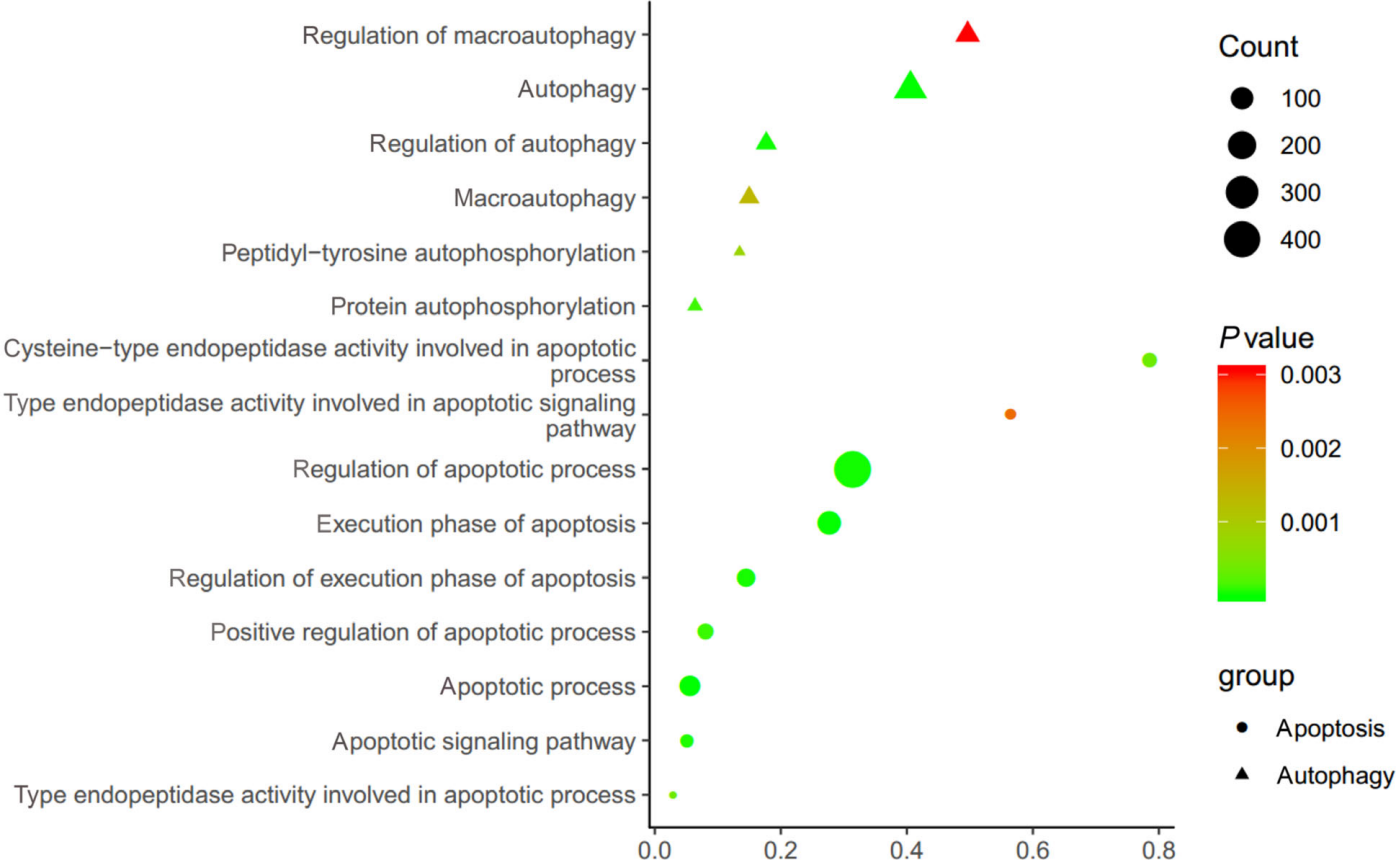
GO analysis. Ranking of top 15 biological processes based on GO analysis of differentially expressed lncRNAs. The bar plot shows the top enrichment score of the significant pathway. The intensity of the color indicates the level of difference; darker shades represent greater difference. The circle represents the relationship; larger circles represent a stronger relationship between the gene and the pathway.

We performed GO enrichment analysis for the differentially expressed genes (DEGs), with particular attention to GO biological processes and molecular function. According to GO analysis, the DEGs were mainly involved in the regulation of autophagy and apoptosis (Fig. 3)

**Fig. 4:**
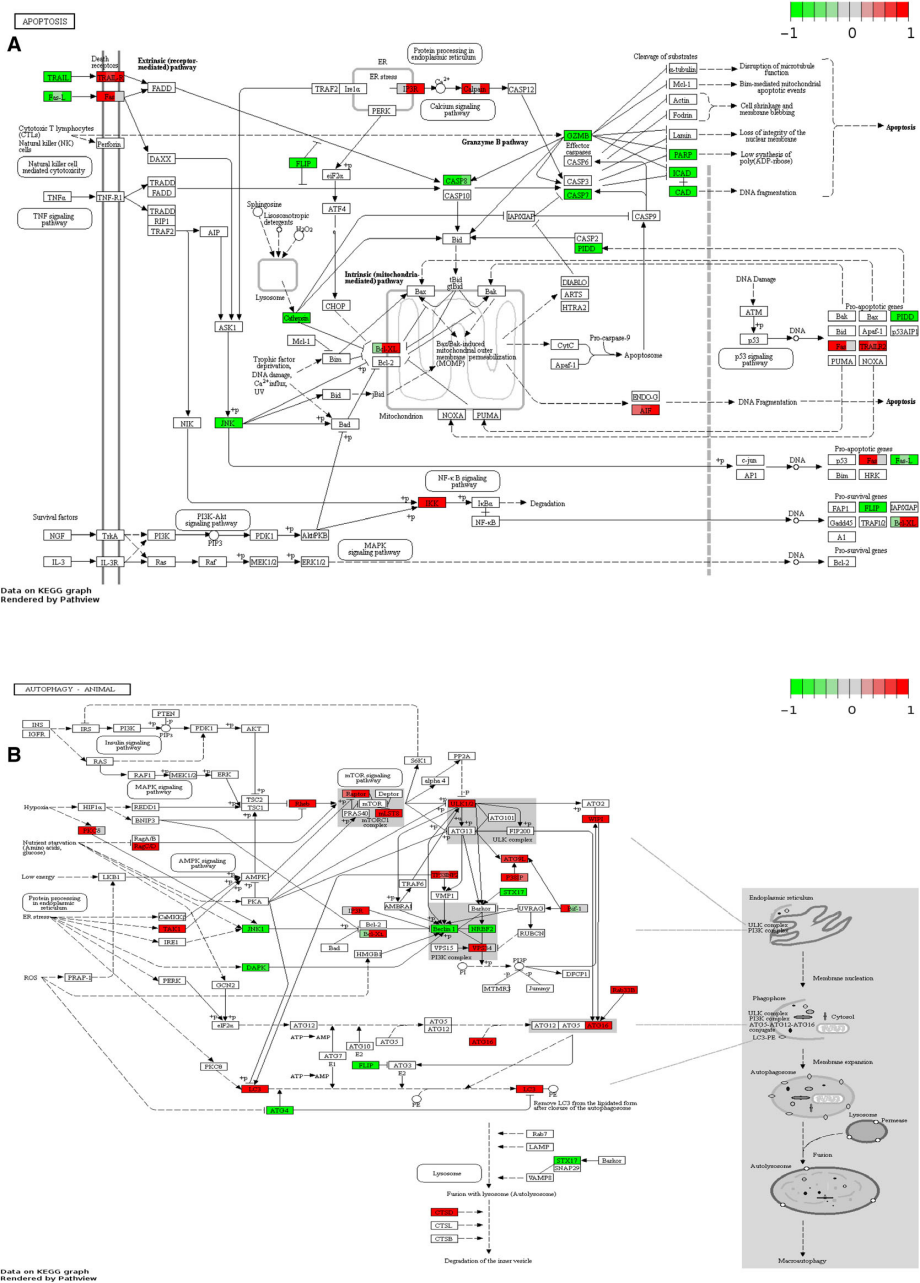
Expression of DEGs involved in the apoptosis and autophagy signaling pathways. (A) Expression of DEGs involved in the apoptosis signaling pathway. (B) Expression of DEGs involved in the autophagy signaling pathway.

Moreover, KEGG pathway analysis showed that these lncRNAs were mainly involved in the nuclear factor‐κB, phosphatidylinositol 3‐kinase (PI3K)‐Akt and Adenosine 5'‐monophosphate (AMP)‐activated protein kinase signaling pathways (Fig. 4A,B)

**Fig. 5:**
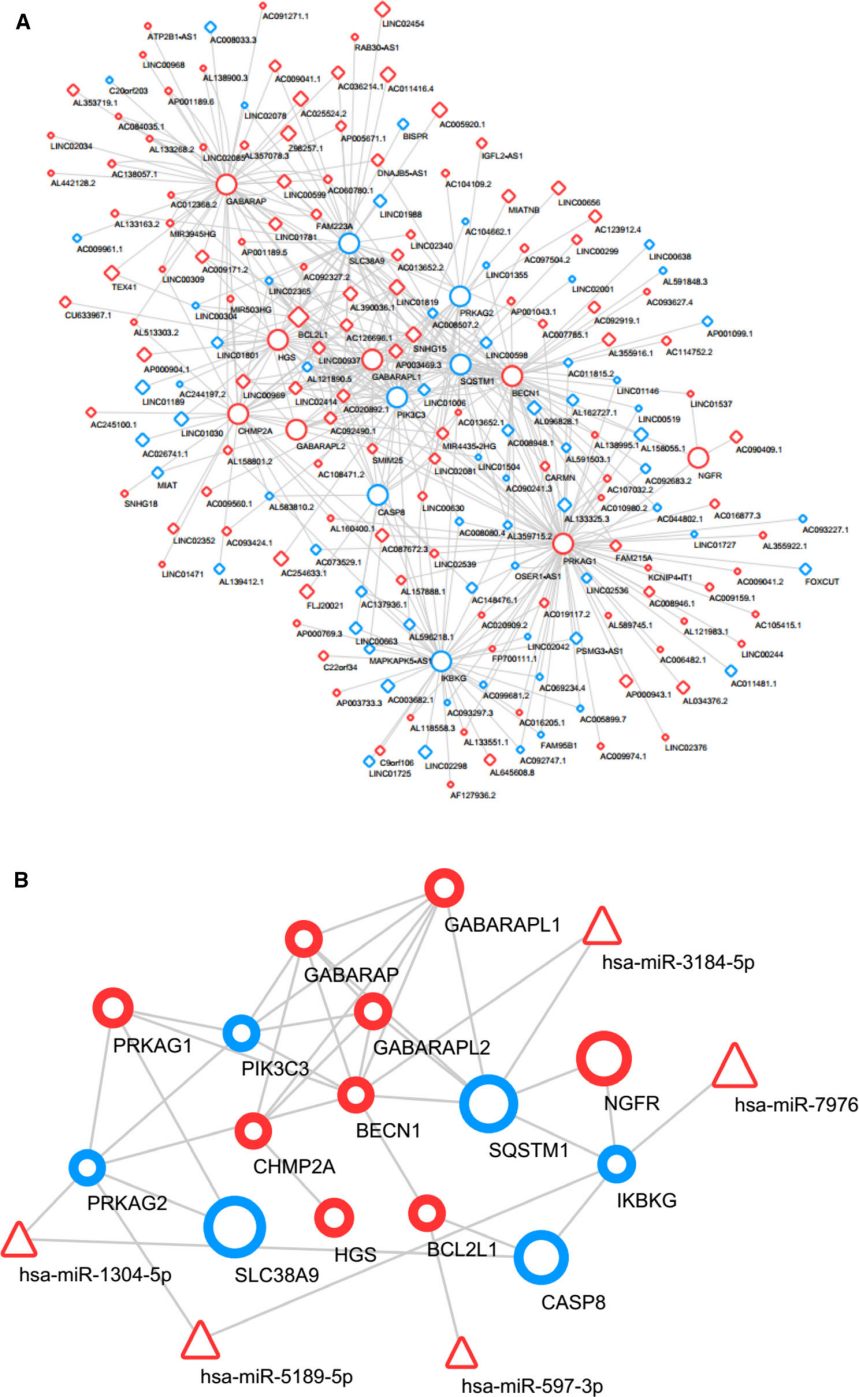
lncRNA–mRNA (A) and miRNA–mRNA (B) interaction networks established based on lncRNA sequences and miRNA target genes. Diamond shapes, triangles and circles represent lncRNAs, miRNAs and mRNAs, respectively. Red represents up‐regulation, and blue represents down‐regulation. Node size represents the degree.

**Fig. 6:**
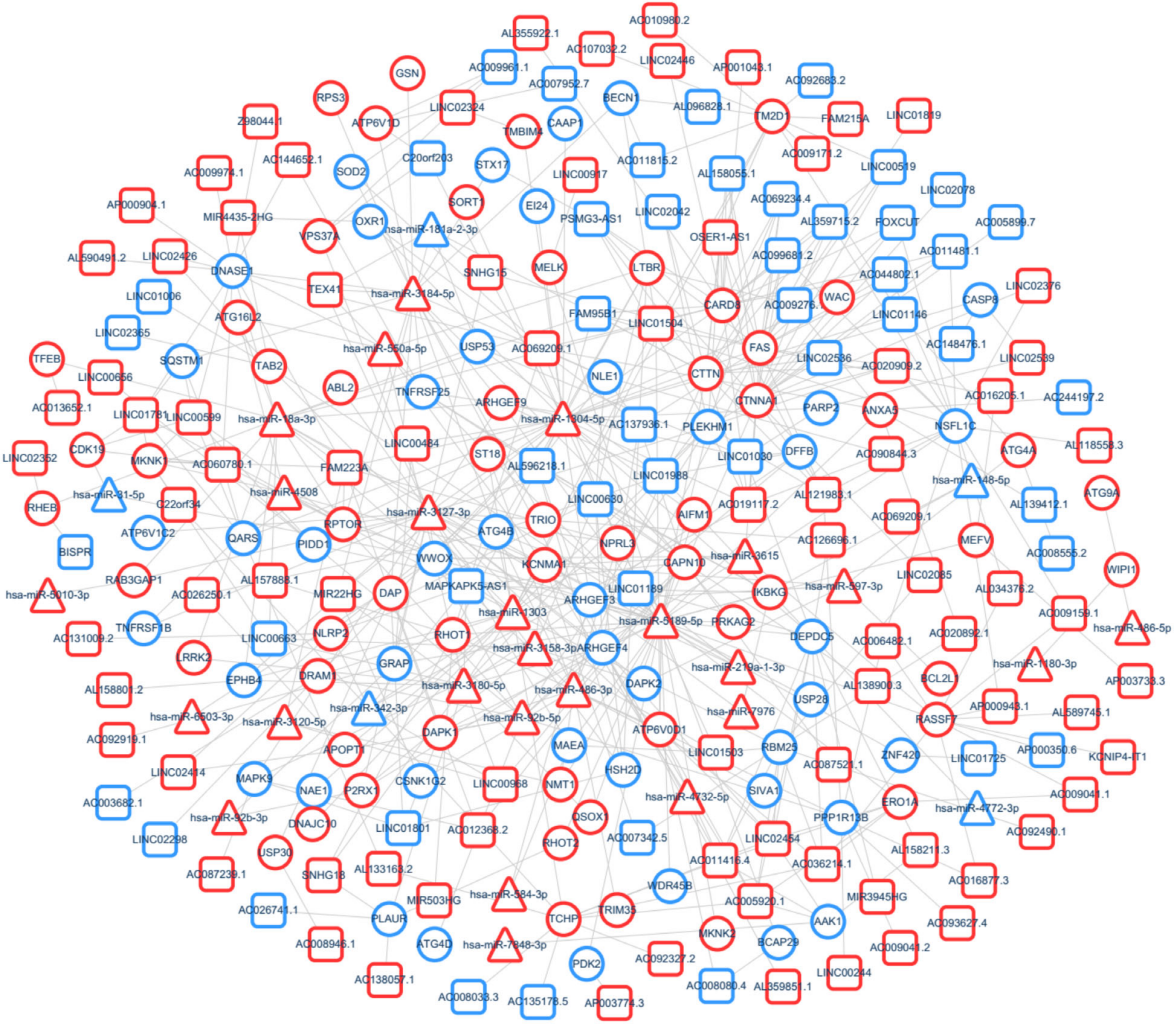
lncRNA–miRNA–mRNA coexpression network. Squares, triangles and circles represent lncRNAs, miRNAs and mRNAs, respectively. Red represents up‐regulation, and blue represents down‐regulation. Node size represents the degree.

To identify the possible modulating mechanisms of the lncRNAs, lncRNA–mRNA and miRNA–mRNA interaction networks were established according to lncRNA sequences and miRNA target genes (Fig. 5A,B), and a lncRNA–miRNA–mRNA co‐expression network was constructed (Fig. 6).

### qRT‐PCR validation of differentially expressed lncRNAs

**Fig. 7:**
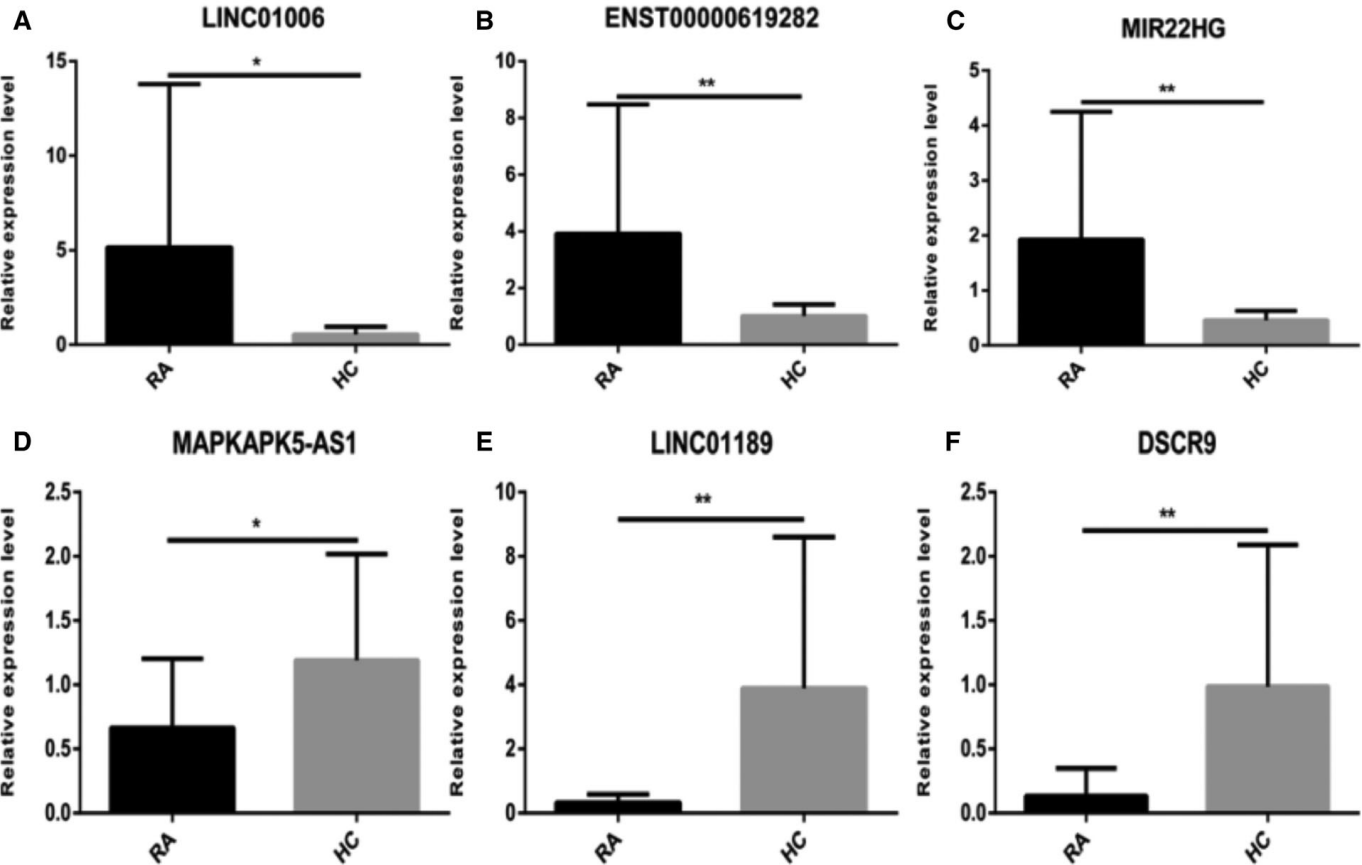
Relative expression levels of lncRNAs. (A–C) The relative expression of ENST00000619282, LINC01006 and MIR22HG was up‐regulated. (D–F) The relative expression of MAPKAPK5‐AS1, LINC01189 and DSCR9 was down‐regulated. All of the results were presented as the mean ± SD based on three replicates involving three samples. The significance difference between two groups was tested via analysis of Student's *t*‐test. *P < 0.05, **P < 0.01, ***P < 0.001.

To validate our RNA sequencing data, we selected two up‐regulated lncRNAs (ENST00000619282 and MIR22HG) and five down‐regulated lncRNAs (MAPKAPK5‐AS1, C5orf17, LINC01189, LINC01006 and DSCR9) related to apoptosis and autophagy. qRT‐PCR analyses were performed to validate candidate gene expression using an independent set of samples from 20 patients with RA and 20 healthy controls. qRT‐PCR showed that the average expression levels of ENST00000619282, LINC01006 and MIR22HG were significantly higher (Fig. 7A–C) and that of MAPKAPK5‐AS1, LINC01189 and DSCR9 were lower (Fig. 7D–F) in the PBMCs of patients with RA as compared with that in the PBMCs of healthy controls. There were no significant differences in the expression levels of C5orf17 between the RA group and the healthy control group.

### Receiver operating characteristic curve analysis of confirmed PMBC lncRNAs of patients with RA

**Fig. 8:**
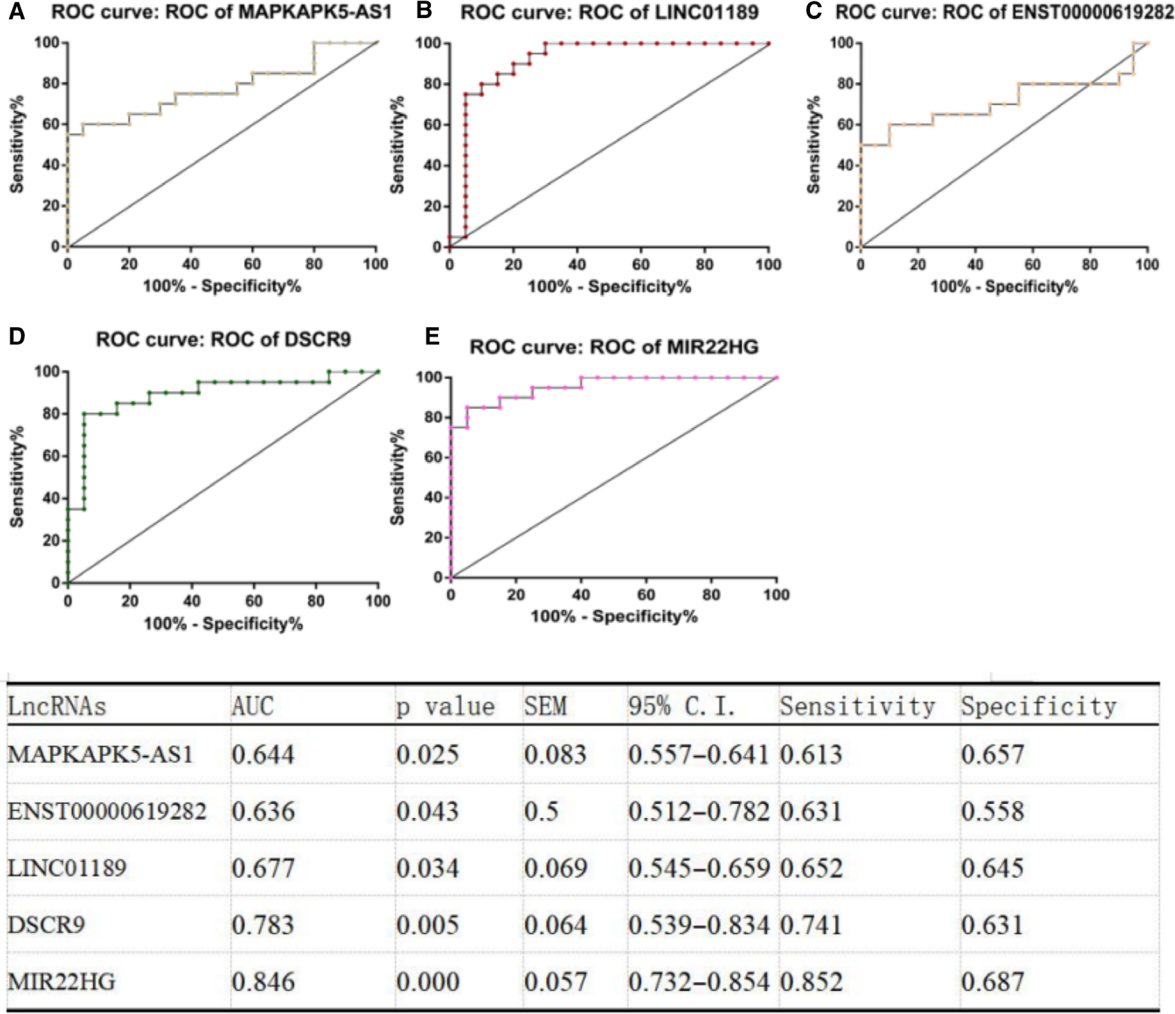
ROC curve analysis of lncRNAs: (A) MIR22HG, (B) DSCR9, (C) LINC01189, (D) MAPKAPK5‐AS1 and (E) ENST00000619282.

Receiver operating characteristic (ROC) curve analysis was performed to evaluate the biological functions and diagnostic value of the four lncRNAs. ROC curves of confirmed lncRNAs showed that the levels of MIR22HG [area under the curve (AUC) = 0.846, *P* = 0.000], DSCR9 (AUC = 0.783, *P* = 0.005), LINC01189 (AUC = 0.677, *P* = 0.034), MAPKAPK5‐AS1 (AUC = 0.644, *P* = 0.025) and ENST00000619282 (AUC = 0.636, *P* = 0.043), as shown in Fig. 8A–E.

### Spearman's correlation test of clinical indexes, SPP and confirmed lncRNAs in PBMCs of patients with RA

**Fig. 9:**
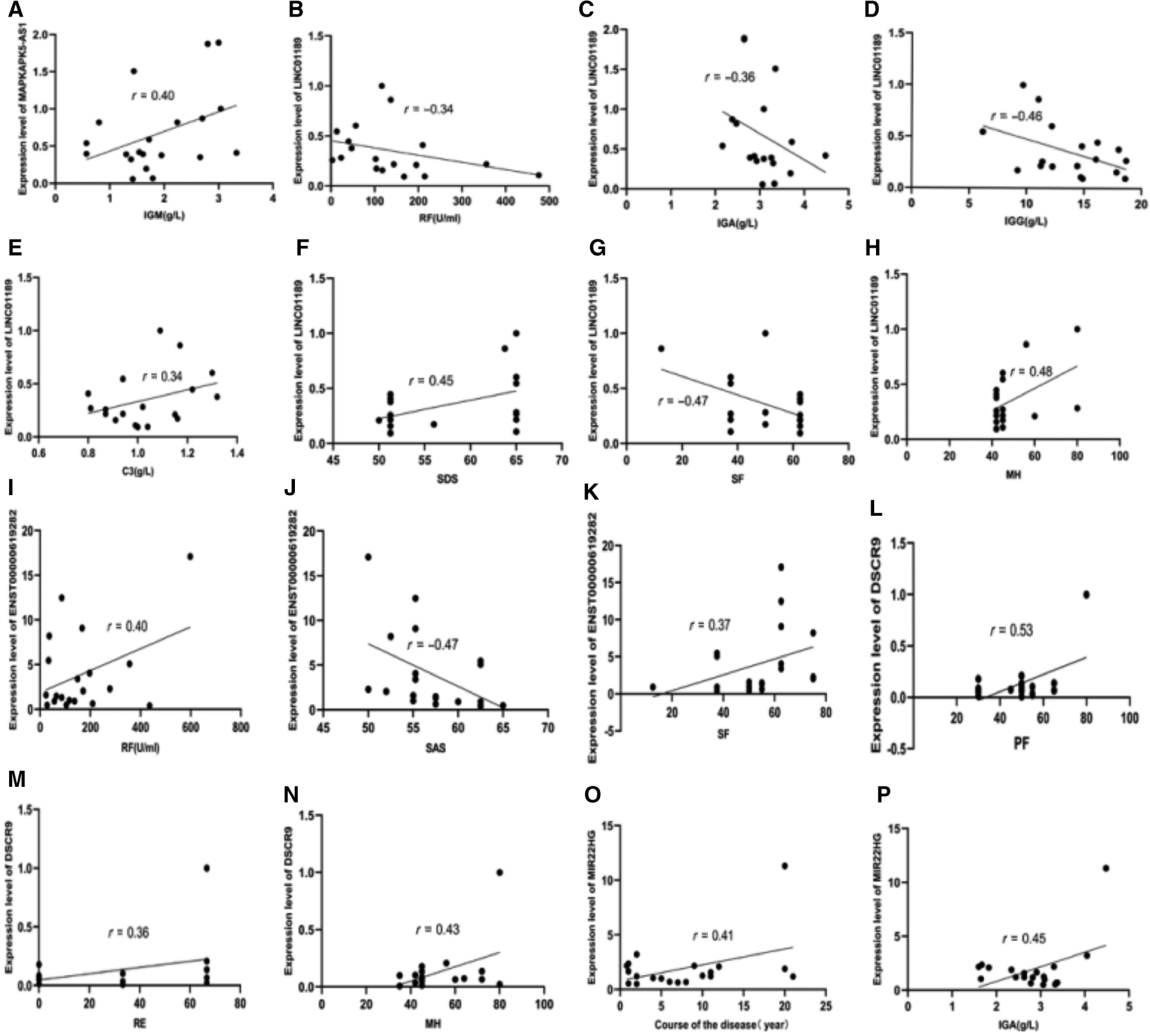
Correlation of lncRNAs with clinical indices and SPP. (A) Correlation between MAPKAPK5‐AS1 and IgM. (B–H) Correlation between LINC01189 and C3, SDS, MH, RF, IgA, IgG and SF. (I–K) Correlation between ENST00000619282 and RF, SF and SAS. (L, M, N) Correlation between DSCR9 and RF, RE and MH. (O, P) Correlation between MIR22HG and the course of disease, IgG.

To determine whether the differentially expressed lncRNAs related to inflammation and immunity in the PBMCs of patients with RA were relevant biomarkers for the severity of RA, we performed the Spearman's correlation test to assess the correlation of MIR22HG, DSCR9, LINC01189, MAPKAPK5‐AS1 and ENST00000619282 with clinical indexes and SPP. Results of the correlation analysis revealed a positive correlation between MAPKAPK5‐AS1 and IgM (Fig. 9A). LINC01189 positively correlated with C3, SDS and MH (Fig. 9B–D), and negatively correlated with RF, IgA, IgG and SF (Fig. 9E–H). ENST00000619282 positively correlated with RF and SF (Fig. 9I,J), and negatively correlated with SAS (Fig. 9K). DSCR9 positively correlated with RF, RE and MH (Fig. 9L–N). MIR22HG positively correlated with the course of disease and IgG (Fig. 9O,P).

## Discussion

lncRNAs are important competing endogenous RNAs [24]. They are a class of noncoding RNAs that regulate diverse physiological processes by controlling gene expression [25, 26]. Intensive studies have been carried out to elucidate the relationship between lncRNAs and diseases, especially cancer [27]. Researchers have discovered many lncRNAs that are considered as potential diagnostic biomarkers of cancers. For example, the lncRNA LINC00173 enhances triple‐negative breast cancer progression by suppressing miR‐490‐3p expression [28]. The lncRNA NORAD regulates lung cancer cell proliferation, apoptosis, migration and invasion via the miR‐30a‐5p/ADAM19 axis [9]. So far, two case reports have shown that the expression of lncRNAs is significantly dysregulated in the PBMCs of patients with RA as compared with that in healthy control PBMCs, which suggests that lncRNAs have the potential to serve as biomarkers of RA [22, 29]. Therefore, changes in the expression of lncRNAs may reflect various underlying diseases.

In this study, we revealed that lncRNAs related to apoptosis and autophagy were differentially expressed in the PBMCs of patients with RA as compared with healthy controls. A total of 341 differentially expressed lncRNAs were detected, of which 231 were up‐regulated and 110 were down‐regulated. Several lncRNAs related to apoptosis and autophagy were aberrantly expressed in patients with RA, suggesting that these lncRNAs may play an important role in the pathogenesis of RA. According to GO analysis, these genes were mainly involved in the regulation of autophagy and apoptosis. Moreover, KEGG pathway analysis showed that these lncRNAs were mainly involved in the nuclear factor‐κB, PI3K‐Akt and Adenosine 5'‐monophosphate (AMP)‐activated protein kinase signaling pathways.

RA is an inflammatory autoimmune disease, in which the immune system of the body mistakenly attacks the joints and other organs [30, 31]. RA affects the flexibility or extensibility of the joints, as well as organ function. The function of lncRNAs in RA provides new insights into the understanding of RA [32]. However, little is known about the diagnostic value of lncRNAs in RA [33]. lncRNAs remove the inhibitory effect of miRNAs on their target mRNAs, indirectly up‐regulating these target mRNAs [34].

In this study, we found that the average expression levels of ENST00000619282, LINC01006 and MIR22HG in the PBMCs of patients with RA were significantly higher than that in the PBMCs of healthy controls (Fig. 7A–C), whereas the expression of MAPKAPK5‐AS1, LINC01189 and DSCR9 was significantly lower in patients with RA as compared with healthy controls. Among these lncRNAs, MIR22HG, DSCR9, LINC01189, MAPKAPK5‐AS1 and ENST00000619282 showed higher ROC AUC and may serve as potential diagnostic biomarkers of RA. In addition, the Spearman's correlation test showed that MAPKAPK5‐AS1 positively correlated with IgM; LINC01189 positively correlated with C3, SDS and MH, and negatively correlated with RF, IgA, IgG and SF; ENST00000619282 positively correlated with RF and SF, and negatively correlated with SAS; DSCR9 positively correlated with RF, RE and MH; and MIR22HG positively correlated with the course of disease and IgG. These results suggest that these lncRNAs are of great importance for practical applications.

However, there are certain limitations to the current study that must be addressed in future research. For example, no significant differences in the expression levels of C5orf17 were observed between the RA and healthy control groups, and C5orf17 had no correlation with clinical indexes and SPP. This discrepancy probably arose because of the relatively small sample size. In addition, the individual differences between patients with RA, including socioeconomic status, disease severity and disease duration, could probably affect the accuracy of the results. Understanding the exact mechanism of lncRNAs in apoptosis and autophagy will be the goal of our next study.

## Conflict of interest

The authors declare no conflict of interest.

## Author contributions

J. Wen, JL, HJ, LW, Yue Sun and LX contributed to the study design. J Wen analyzed the data, wrote the first draft and revised the manuscript. Yanqui Sun, YZ and PZ contributed to specimen and data collection. XD, XW and J. Wang supervised the project and contributed to manuscript revision. All authors reviewed and accepted the content of the final manuscript.

## Data Availability

The data used to support the findings of this study are available from the corresponding author upon request.
